# An epidemiological survey of porcine cysticercosis in Nyasa District, Ruvuma Region, Tanzania

**DOI:** 10.1016/j.parepi.2017.09.002

**Published:** 2017-09-29

**Authors:** Seria M. Shonyela, Ernatus M. Mkupasi, Sikasunge C. Sikalizyo, Evance M. Kabemba, Helena A. Ngowi, Isaac Phiri

**Affiliations:** a P.O. BOX 14 Songea, Ruvuma, Tanzania; b Department of Vet. Med. & Public Health, Sokoine University of Agriculture, P.O. BOX 3000 Morogoro, Tanzania; c Department of Para-clinical Studies School of Veterinary Medicine, University of Zambia, P.O. BOX 32379 Lusaka, Zambia

**Keywords:** *Taenia solium* cysticercosis, Prevalence, Risk factors, Pig management, Nyasa, Tanzania

## Abstract

Porcine cysticercosis (PC) caused by *Taenia solium* larvae is continuing being important zoonotic disease in many developing countries. It poses a serious public health risk and leads to economic losses to pig production industry. This study was carried out to determine the prevalence and risk factors associated with PC transmission in Nyasa District. To establish the prevalence of PC, a cross-sectional survey was conducted involving 698 pigs by tongue examination, 330 pigs by Ag-ELISA test and 22 pigs by meat inspection. A questionnaire survey was administered to a member of selected households to gather information on pig management and other potential factors that could explain the prevalence of PC in the area. Results showed that 44 pigs were positive by tongue examination (6.3%, 95% C.I. 4.5–8.1%), 110 tested positive for Ag-ELISA (33.3%, 95% C.I. 28.22–38.38%) and meat inspection detected four infected pigs (18.2%, 95% C.I. 2.08–34.32%). Risk factors associated with PC transmission in Nyasa District were free ranging of pigs (*p* = 0001), sex of pig (*p* = 0.011), source of pork (*p* = 0.0001) and outdoor defecation (0.0001). The present findings indicate that PC is endemic in Nyasa District and that free-ranging of pigs in conjunction with limited use of latrines contributes significantly to PC transmission. Therefore, mandatory pig confinement, together with use of latrine/toilets should be considered in controlling PC in Nyasa District.

## 1 Introduction

Pig production is an important economic activity to many poor families in developing countries (Phiri et al., 2003, Karimuribo et al., 2011). However, *T. solium* cysticercosis is being reported in these countries resulting into both agricultural and public health impacts (Eom et al., 2009, Phiri et al., 2003, Zoli et al., 2003, Ngowi et al., 2013). The parasite causes taeniosis in human, who is the definitive host and cysticercosis in pigs which are principal intermediate hosts. Human being acquires taeniosis following ingestion of raw or undercooked pork infected with viable *T. solium* cysticerci (Murrell, 2005). Human being also can develop cysticercosis following accidental ingestion of *T. solium* eggs. Lodging of cysticerci of *T. solium* in the brain results in neurocysticercosis (NCC), one of the most important neurological parasitoses in human and the main preventable cause of acquired epilepsy in endemic areas (Carabin et al., 2011). Neurocysticercosis is now recognised as an important public health problem in both developing and developed countries (White, 2000).

Porcine cysticercosis has been reported in many sub-Saharan countries with prevalence rates as high as 64% (Dorny et al., 2004). In East African countries, prevalence of cysticercosis infection among pigs in a number of areas reported to be approximately 20% (Phiri et al., 2003, Ngowi et al., 2004). A study in few villages of Ruvuma rural and Mbinga Districts in Ruvuma region Tanzania estimated the prevalence of PC to be 16.9% (Boa et al., 2006a, Boa et al., 2006b). Endemicity of *T. solium* cysticercosis in developing countries have been associated with general poverty, free ranging of pigs, poor sanitary conditions and poor knowledge about the parasite (Phiri et al., 2003, Murrell, 2005, Sikasunge et al., 2007). In addition, home slaughter of pigs without inspection, inadequate pork cooking before consumption were also found to be responsible for the reported prevalence (Boa et al., 2006a, Boa et al., 2006b).

In Nyasa District pig keeping is common and free range type of pig management is practiced, however, no study has been conducted to establish the status of PC and risk factors for its transmission. This study investigated the prevalence of porcine cysticercosis and risk factors for *T. solium* cysticercosis transmission in Nyasa District. The information is useful in planning parasite control measures in the area to promote socio-economic development through pig production.

## 2 Materials and methods

### 2.1 Study area

**Fig. 1:**
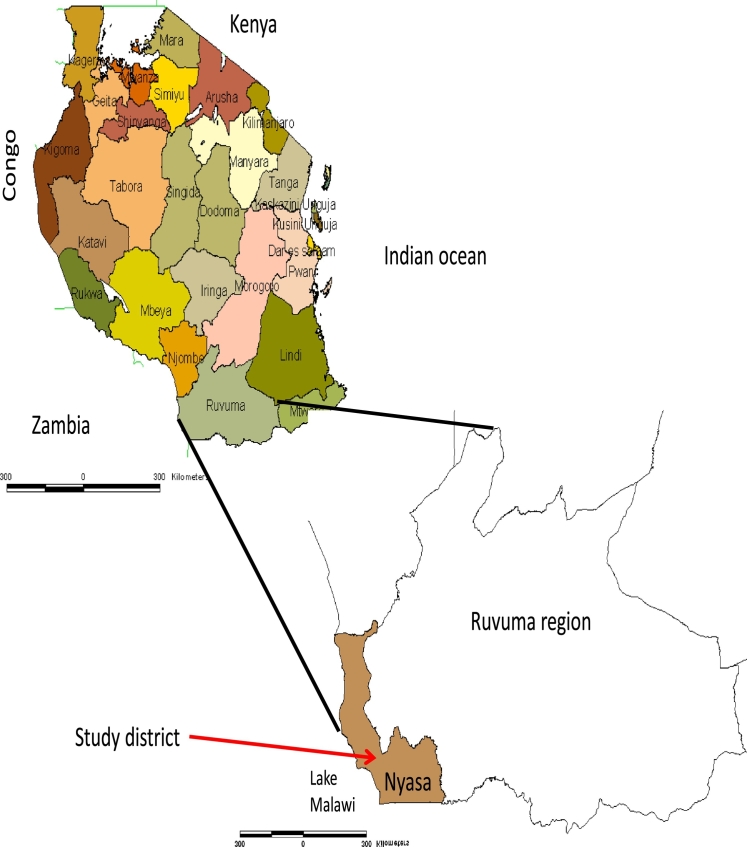
Map of Tanzania (top) and Ruvuma region with pointed study area (bottom)

This study was conducted in Nyasa District, Tanzania (Fig. 1) in the period of September 2013 to January 2014. The study area was selected due to an observed free range pig husbandry and that no previous study on porcine cysticercosis has been conducted in the area. Nyasa District is one of the six administrative districts of Ruvuma region. The District is located between latitude 10° 15′S and 11° 34′N and Longitude 35° 24′ E and 34° 28′ W with an area of 3,811 km^2^. Administratively the District is divided in three divisions namely; Ruhuhu, Ruhekei and Mpepo with human population of 146,160 (NBS, 2013). Major economic activities in the district were subsistence farming with fishing and mining on a small scale. Pig population estimated to 138,568 for Mbinga District (NSCA, 2012), which later divided to Mbinga and Nyasa Districts.

### 2.2 Study design and sample size estimation

A cross-sectional survey was carried out to determine the prevalence *T. solium* cysticercosis and associated risk factors. Multistage sampling strategy was adopted whereby the three divisions of the District were included. In each division, four villages keeping pigs were randomly selected and all pig keeping households in the selected villages were sampled. Pig keeping households were established with the help of livestock extension officer and local administration. Sample size for pigs was computed using the formula *n* = *Z*^2^*PQ*/*L*^2^ (Martin et al., 1987). Where, n is the required sample size, Zα = 1.96 is the standard normal deviate at 5% level of significance, p is the estimated prevalence, q = 1-p, and L is the precision of the estimate. Setting *p* = 0.169 (Boa et al., 2006a, Boa et al., 2006b) and L at 5%, the required sample size was 216 pigs. In households with more than two pigs, a proportion of the pigs were randomly selected for sampling. For instance where there were three pigs two were sampled and where there were five, three were sampled.

### 2.3 Data collection

Consent was sought from pig owners before sampling and samples were only collected from those who accepted to participate in the study. Clinically healthy pigs, older than two months and neither pregnant nor suckling sows were sampled. For tongue examination, a pig was firmly restrained and pig snare was used to stabilize the head and hard wooden stick used to open the mouth. Using a piece of cotton gauze for grip, tongue was gently pulled out, examined and palpated all along its ventral surface and base for presence of cysticerci. Pigs were considered positive for cysticercosis if cyst-like nodules were either seen or felt. Tongue examined pigs were bled to obtain about 5 ml of blood from the anterior vena cava using plan vacutainers. The collected blood was transported in cool box to the laboratory and kept at 4 °C overnight and then centrifuged at 3000*g* for 15 min. The supernatant (serum) was aliquoted in well labelled 1.8 ml cryogenic vials and stored at − 20 °C until use.

#### 2.3.1 Meat inspection

Meat inspection of slaughtered pigs was carried out in two official slaughter slabs located at Mbambabay and Lituhi in the District. Routine meat inspection was conducted to assess the presence of *T. solium* cysticerci in the masseter, triceps brachial, tongue, psoas and heart muscles. Two longitudinal incisions were made into the external masseter muscles and one into internal masseter muscles on both sides of the lower jaw and presence of cysticerci was noted. The tongue was removed from the head, the surface visually inspected and palpated then a deep longitudinal incision was made on the ventral surface, extending the whole length of the tongue and examined.

#### 2.3.2 Risk factors associated with porcine cysticercosis transmission

A structured questionnaire with both closed and open ended questions was administered to a member of selected household who was familiar with the day to day raising of pigs owned by that household. Questionnaire was pretested by administration to ten individuals from pig keeping households selected from two divisions in nearby Mbinga District. Questionnaire was written in English, given that majority of respondents could not understand English, the questionnaire was translated and administered in Swahili. The questionnaire survey obtained data on pig production and risk factors for occurrence of porcine cysticercosis. Risk factors considered in this study included division of origin, pigs husbandry system, sex of pigs, age of pigs, presence and lack of evidence of latrine use, open defecation, pork preparation, sources of pork, home slaughter, lack of pork inspection and community awareness on porcine cysticercosis.

#### 2.3.3 Laboratory sample analysis

Serum samples were examined for the presence of circulating cysticerci antigens using a double monoclonal antigen-based sandwich Ag-ELISA as described by Dorny et al. (2000) with minor modifications (Sikasunge et al., 2007). Briefly, two monoclonal antibodies (MoAb) used in the ELISA were B158C11A10 diluted at 11.8 mg/ml in carbonate buffer (0.06 M, pH 9.6) for coating and a biotinylated MoAb B60H8A4 diluted at 3.2 mg/ml in phosphate buffered saline-Tween 20 (PBS-T20) + 1% new born calf serum (NBCS) as detector antibody. Streptavidinhorseradish peroxidase (Jackson Immunoresearch Lab, Inc.) diluted at 1/10,000 in PBS-T20/1% NBCS was added to act as conjugate. The plates were read and delta optical densities were calculated.

### 2.4 Data analysis

SPSS version 16 was used for statistical data analysis. Descriptive statistics were used to compute the prevalence while associations between the potential risk factors for tongue examination and Ag ELISA results were assessed using the chi-square statistic, and strengths of associations were determined using the odds ratio (OR). Predictors of porcine cysticercosis transmission were computed using forward step-wise binary logistic regression model. Risk factors with *P* < 0.005 were considered to be significant.

## 3 Results

### 3.1 General descriptions

A total of 698 pigs were investigated in this study by using tongue examination. Of the total number of pigs examined by tongue examination, blood was collected from 330 pigs while meat inspection was done on 22 pigs. Of the 698 pigs examined on tongue examination, 44 (6.3%) were positive for cysticercosis. Out of the 330 pig sera analysed 110 (33.3%) were positive for cysticercosis by Ag-ELISA and of the 22 carcasses inspected 4 (18.2%) were positive for cysticercosis. Structured questionnaires were administered to individuals in 284 households in the selected villages including both pig keepers and non-pig keepers. Of the respondents 73.2% were males. Free range management of pigs found to be common in the study area (67%) and about 95% of respondents reported to consume pork.

### 3.2 Prevalence of porcine cysticercosis in Nyasa District

The positive pigs based on tongue examination and Ag - ELISA per division were as presented in Table 1. Meat inspection was conducted in two official slaughter places where four out of 22 (18.2%) inspected pig carcasses were positive.

**Table 1:** Prevalence of porcine cysticercosis by Tongue and Ag ELISA tests by division.

Division	Tongue examination				Ag ELISA			
	Pigs exam	Positive pigs	Prevalence (%)	95% C.I.	Pigs exam	Positive pigs	Prevalence (%)	95% C.I.
Ruhuhu	335	29	8.7	5.68–11.72	232	49	21.1	15.8–26.35
Ruhekei	135	8	5.9	1.93–9.87	57	40	70.2	58.3–82.07
Mpepo	228	7	3.1	0.85–5.35	41	21	51.2	35.9–66.5
Total	698	44	6.3	4.5–8.1	330	110	33.3	28.22–38.38

C.I. – confidence interval.

### 3.3 Risk factors associated with porcine cysticercosis transmission in the District

Risk factors significantly associated with porcine cysticercosis transmission following univariate analysis of the hypothesized factors are presented in Table 2., and predictors of porcine cysticercosis on Ag-ELISA by forward step-wise binary logistic regression model on Table 3.

**Table 2:** Risk factors associated with porcine cysticercosis transmission in Nyasa District.

Factor	Level	Pigs examined	Positive pigs	Prevalence	*P*-value
Sex	Male	62	11	17.7%	0.011
	Female	268	99	36.9%	
Pig husbandry	Free range	179	95	53.1%	0.001
	Confined	151	15	9.9%	
Outdoor defecation	Yes	120	23	19.2	0.0001
	No	210	87	41.4	
Source of pork	Butcher	207	92	44.4	0.0001
	Ceremonies	116	16	13.8

**Table 3:** Predictors of porcine cysticercosis on Ag-ELISA using forward step-wise binary logistic regression model.

Predictors	Point or rank	Odds ratio	(95% CI)	P-value
Pig husbandry	Free range/Tethering	10.925	5.539 to 21.549	0.000
	Confined	Ref		
Sex	Female	0.247	0.102 to 0.599	0.002
	Male	Ref		
Divisions	Ruhekei	0.207	0.091 to 0.472	0.000
	Mpepo	2.423	0.870 to 6.750,	0.09
	Ruhuhu	Ref		

## 4 Discussion

This study investigated the prevalence and risk factors associated with PC transmission in Nyasa District. Findings show that PC is prevalent in the study area this demonstrates the existence of favorable conditions for complete life cycle of the parasite to occur.

High PC sero-prevalence found in this study is comparable to what reported in some countries in sub-Saharan Africa. For instance, a study in Angónia District of Tete Province in North-western Mozambique reported a sero-prevalence of 34.9% (Pondja et al., 2010). However, the findings of this study were higher than what reported by Sikasunge et al. (2008) in Zambia and Ngowi et al. (2004) in Tanzania. The observed variations might reflect the differences in level of environmental contamination in the study areas.

Prevalence of PC found in this study by tongue examination was also comparable to what reported in a previous study conducted in the same zone which was 7.6% and 8.4% for Chunya, and Iringa Rural Districts, respectively (Boa et al., 2006a, Boa et al., 2006b). The slightly lower prevalence in this study could be due to the outbreaks of African swine fever in the years of 2010–2011 as many pig farmers started to confine their pigs in fear of contracting the disease. There were also some sanitation promotion programmes which included pig confinement in the nearby Districts (Mbinga and Ludewa) in which the impact might have spilt over to communities in the study District. Though having low sensitivity, tongue examination method is useful for screening infected pigs in the field being quick and inexpensive. The lower prevalence estimated by tongue examination compared to Ag-ELISA test could probably be due to the reported manipulation of tongue cysts as it was revealed by some farmers in the study area. The same scenario has been reported from other parts of the country (Komba et al., 2013). Also could be explained by the fact that sometimes cysticerci will not reach the tongue and also requires more than two months to develop to visible cysticerci hence immature ones are likely to be missed (Garcia et al., 2003). A pig diagnosed positive by traders reported to be rejected and taken back to the owner. There is no proper monitoring of such cases hence infected pork may enter the food chain leading to more human infections if preparation is inadequate. For effective control of the infection, monitoring of rejected infected pigs at farm level should be instituted to interrupt the life cycle of the parasite.

Furthermore the prevalence of PC on post-mortem examination in this study was also comparable with results obtained in previous study conducted in neighbouring Mbinga District (Boa et al., 2006a, Boa et al., 2006b). However, small number of pigs examined in this study makes comparison of the two studies difficult. Low number of infected pigs encountered in this study partly could be explained by the fact that most farmers and traders are increasingly becoming aware on the economic consequences of the disease when encountered at official slaughter places. Therefore, traders screen pigs by tongue examination at the farm and exclude infected ones. Similarly, a study conducted by Coulibaly and Yameogo (2000) in Burkina Faso recorded low prevalence (0.57%) of PC based on meat inspection reports. Another important observation from this study is the presence of few official pig slaughter places. This implies that home and communal congregation areas such as local brew clubs slaughtering of pigs is practiced where inspection is rarely done as also revealed by some respondents. This might be an important risk factor for transmission of the infection in the study area.

In reference to location, variation in prevalence among the three divisions sampled in the District was observed (Table 1), with the highest prevalence by Ag-ELISA being Ruhekei division and the difference was statistically significant (*p* < 0.001). The cause for the observed dissimilarity are likely to be variations in pig management practice where in Ruhekei pigs reported to be more free ranged as compared to Mpepo and Ruhuhu divisions. Pig management practice varied depending to the season; during rainy season when crops were still in fields pigs were kept indoors or tethered while during dry season were left free to scavenge. However, tethering was in our view not a protective way of preventing pigs from getting infected with porcine cysticercosis as it was carried out on the grazing land or on the bushes where people used to defecate. Sows are usually tethered while the piglets were left roaming freely. This finding could probably explain why there was no significant difference between free ranging and tethering methods as pigs could possibly access human faeces containing the parasite eggs.

In this study it was also observed that even the intensively kept pigs were fed on grasses, cassava leaves, cassava roots, and other plant material harvested from bushes where possible human faecal contamination could occur. One of the reasons why farmers practice free range and tethering methods of pig keeping was the financial constraint to meet the costs associated with pig confinement. This is in agreement with Gilman et al. (1999) who reported that a pigs can be kept cheaply by allowing them to roam free in villages thereby obtain a variety of food to supplement their diet. Therefore unless the feeding challenge is addressed, free ranging of pigs in the study area will continue risking them from acquiring PC.

Regarding to sex, female pigs had significantly higher (*p* = 0.002) prevalence compared to male pigs by Ag-ELISA (Table 3). This observation is similar to what reported by Khaing et al. (2015) in a study conducted in Myanamar. It can be explained that female pigs were kept longer for breeding purposes than males increasing risk of being exposed to *T. solium* eggs (Khaing et al., 2015). Another possible reason for the observed difference in prevalence could be due to the large number of female pigs (sample bias) examined in this study. On the other hand, results from this study differ from what reported from studies conducted in Zambia (Sikasunge, 2005) and in Mexico (Garcia et al., 2003) who reported higher prevalence in males than in female pigs. But Jayashi et al. (2012) reported that sex was not a significant risk factor for PC. From these observations, we can conclude that sex might not have an influence to the infection to occur rather the duration of exposure to the infection.

The present study demonstrated lack of association between age and prevalence of porcine cysticercosis by Ag ELISA, although tongue examination indicated that as age of pig increases also the risk of being positive to *T. solium* cysticercosis increases (Table 1). This finding could be explained by the fact that adult pigs have long exposure time to the environment that could be contaminated with human faeces. The observed lack of association on Ag-ELISA is in agreement with the findings reported by Sikasunge et al. (2008) in Zambia, Sarti et al. (1992) in Mexico and Pouedet et al. (2002) in Cameroon. Similarly, in some studies lack of association between age and prevalence of infection with PC has been reported (Sakai et al., 1998, Nguekam, 1998). It has been found that pigs in endemic areas are infected early in their lives; however, it requires about three months to develop to visible cysticerci but can be detected by Ag ELISA as early as two weeks following exposure to the infection.

Although latrine coverage in study area was good, direct observation and response from some respondents revealed poor latrine use (Table 4). Respondents in this study revealed that they shunned using latrines because of foul odours, flies, latrine deluging and lack of privacy as also reported by Rajshekhar et al. (2003). Furthermore, in most rural communities children are discouraged to use toilet/latrine due to danger of falling into a pit or drowning (Phaswana-Mafuya, 2006). This implied that a good number of household members practice outdoor defecation. This practice could be playing a big role in transmission of PC in the study area. Eshitera et al. (2012) in a study conducted in Kenya found a significant relationship between lack of latrines use and PC prevalence. Many studies also have shown that lack of latrines and or limited usage is a risk factor for PC (Ngowi et al., 2004, Sikasunge et al., 2007, Eshitera et al., 2012). Interestingly some respondents reported that they constructed toilets for fear of being fined by the authorities in case they don't have at their homes. Thus there was a tendency of many people in the study area to defecate in farms, bushes or in close proximity to pig pens. Based on this observation, it is important that communities should be sensitized on the need to have and use toilets.

**Table 4:** Questionnaire results on pig management practices and sanitation in Nyasa District.

Variables		# of people responded	Percentage (%)
Factor	Management		
Pig husbandry	Free range/tethering	189	66.5
	Confined	95	33.5
Presence of toilet	Yes	282	99.3
	No	2	0.7
Outdoor defecation	Yes	76	26.8
	No	208	73.2
Pork consumption	Yes	275	96.5
	No	9	3.2
Source of pig meat	Butcher	193	70.2
	Funerals/ceremony	82	29.8
Means of cooking pork	Boiling	119	43.3
	Roasting/frying	131	47.6
	Barbecuing	25	9.1

# - Total number.

Community awareness on *T. solium* cysticercosis- taeniasis complex is key towards its eradication (Ngowi et al., 2008, Mafojane et al., 2003). Results from questionnaire study showed that majority of respondents (80.3%) had neither heard about PC nor seen pork with cysticerci (Table 4). This observation support earlier findings by Boa et al. (2001) in some areas of the southern highlands of Tanzania and Shey-Njila et al. (2003) in North West Cameroon that there is lack of knowledge about biology of the parasite in most endemic areas. Limited knowledge led to wrong community perception about transmission of *T. solium*. Most of the participants thought that PC was caused by feeding cassava leaves or by mating a cysticercosis infected pig with a non infected one. Due to incomplete knowledge on life cycle of *T. solium*, in the District, farmers practiced activities which favoured perpetuation of the infection within the area such as; defecating in the bush, consumption of infected pork and free ranging of pigs.

## 5 Conclusion and recommendation

*Taenia solium* cysticercosis is still a serious agricultural and public health problem in the study area. High prevalence of PC in the study area suggests the existence of favorable conditions for full life cycle of the parasite to occur. In spite of good coverage of toilets in the study area, the use is poor. This study also revealed that farmers lacked knowledge of *T. solium* and its life cycle. Hence, for effective control of the parasite public health education should be included. Education should also include good pig management practice to minimize risk of pigs acquiring the infection and improve the welfare of pigs. Local by laws should be formulated and enforced to control pig management and construction and use of latrines/toilets in the study area. The obtained information will guide future efforts towards control and ultimately elimination of the parasite.
